# Health Equity and Disparities in ROP Care: A Need for Systematic Evaluation

**DOI:** 10.3389/fped.2022.806691

**Published:** 2022-04-01

**Authors:** Tochukwu Ndukwe, Emily Cole, Angelica C. Scanzera, Margaret A. Chervinko, Michael F. Chiang, John Peter Campbell, Robison Vernon Paul Chan

**Affiliations:** ^1^Department of Ophthalmology and Visual Sciences, Illinois Eye and Ear Infirmary, University of Illinois at Chicago, Chicago, IL, United States; ^2^National Institutes of Health, National Eye Institute, Bethesda, MD, United States; ^3^Department of Ophthalmology, Casey Eye Institute, Oregon Health & Science University, Portland, OR, United States

**Keywords:** retinopathy of prematurity (ROP), health equity, disparities, social determinants of health, premature infants

## Abstract

Retinopathy of prematurity (ROP) is a vasoproliferative retinal disorder that can have devastating visual sequelae if not managed appropriately. From an ophthalmology standpoint, ROP care is complex, since it spans multiple care settings and providers, including those in the neonatal intensive care unit (NICU), step down nurseries, and the outpatient clinic setting. This requires coordination and communication between providers, ancillary staff, and most importantly, effective communication with the patient's family members and caregivers. Often, factors related to the social determinants of health play a significant role in effective communication and care coordination with the family, and it is important for ophthalmologists to recognize these risk factors. The aim of this article is to (1) review the literature related to disparities in preterm birth outcomes and infants at risk for ROP; (2) identify barriers to ROP care and appropriate follow up, and (3) describe patient-oriented solutions and future directions for improving ROP care through a health equity lens.

## Introduction

Retinopathy of prematurity (ROP) is a vasoproliferative retinal disorder that can have devastating visual sequelae if not managed appropriately. Retinal examinations at regular intervals performed by a trained ophthalmologist are imperative to diagnose vision-threatening disease, and current treatment options can have excellent outcomes if disease is recognized and treated in a timely fashion. An estimated 400–600 infants in the United States become legally blind from ROP (1), and though there has been an overall increase in ROP screening from 2008 to 2018, ~10% of infants were not screened (2). From an ophthalmology standpoint, ROP care is complex, since it spans multiple care settings and providers, including those in the neonatal intensive care unit (NICU), step down nurseries, and the outpatient clinic setting. This requires coordination and communication between providers, ancillary staff, and most importantly, effective communication with the patient's family members.

There is significant medicolegal liability associated with ROP care, often related to system-wide challenges that result in failure to engage the family, ineffective communication between ancillary staff and family members, failure of care coordination between the inpatient and outpatient setting, and physician factors related to knowledge and skills related to diagnosis and treatment (3). Major risk factors for the development of ROP such as birth weight, gestational age, oxygen use, and maternal and infant factors have been well described (4). However, barriers to ROP follow up and adherence to care have not been systematically evaluated and there is limited literature related to disparities in ROP care and outcomes.

It is often factors related to the social determinants of health that can play a significant role in effective communication and care coordination with the family, and it is important for ophthalmologists to recognize these risk factors. The aim of this article is to (1) review the literature related to disparities in preterm birth outcomes and infants at risk for ROP; (2) identify barriers to ROP care and appropriate follow up, and; (3) describe patient-oriented solutions and future directions for improving ROP care through a health equity lens.

## Methods

A literature search was performed between July and October 2021, including literature published in English between 1992 and 2021. We only excluded articles that were not related to ROP care in the United States. Electronic databases used included PubMed, Scopus, Google Scholar, and Dimensions. PubMed search terms and variants included health services accessibility, health services needs and demands, healthcare disparities, premature birth, quality of healthcare, retinopathy of prematurity, social determinants of health, socioeconomic factors, treatment outcome, and poverty areas. Scopus search criteria included retinopathy of prematurity, low birth weight, socioeconomic and related factors, inequities, disparities, insecurities, economic, income, racism, retina. Other search terms include access to care, financial insecurity, social support networks, social services, community health workers, race, gender, parents, health literacy, language barriers, limited English proficiency, outpatient follow up, transitions of care, and insurance. Variations of these terms were used to ensure exhaustive search results. Our search strategy was supplemented by manual reference searching of relevant article bibliographies and other review articles.

### Disparities in Preterm Births and Retinopathy of Prematurity

Health disparities have been defined by the Centers for Disease Control as preventable differences in the burden of disease, or opportunities to achieve optimal health, that are experienced by populations (5). Understanding and identifying trends in preterm births is essential to identify the characteristics of populations at risk for worse outcomes in ROP that are related to systemic health disparities. From 1971 to 2018, preterm births (PTB) constituted 11% of all births in the United States (6). Determinants of PTB include race, socioeconomic status, history of maternal substance abuse, and maternal health factors (7). There are profound racial disparities in PTB in the United States. In 2019, the rate of preterm birth among Black women (14%) was about 50 percent higher than the rate of preterm birth among White or Hispanic women (9.3 and 10%, respectively) (7, 8). In a study analyzing 2016 U.S. birth certificate data, results found that nearly 38% of the preterm birth disparities were noted between black and white infants (9). Another study found that these differences still existed even among college-educated women with private insurance (10). Even when controlling for socioeconomic risk factors, racial disparities in neonatal outcomes have been shown to exist (11). Although these disparities remain largely unexplained, they are thought to be due to multifaceted risk exposures from generations of socioeconomic disadvantage. It has also been hypothesized that neighborhood deprivation (12–16) and a family history of preterm birth (17) may also play a significant role.

In the United States, there have been noted racial and ethnic variations in ROP. This was explored in the Cryotherapy for Retinopathy of Prematurity (CRYO-ROP) Cooperative group, noting that ROP occurred with similar frequency in all racial subgroups, though severe ROP was less common in Black infants (18). Additionally, it was found that there was an increased risk of reaching threshold ROP associated with lower birth weights, younger gestational age, white race, multiple births, and being born outside a study center nursery (19). Port et al. also noted that black race was found to be a protective factor for treatment-requiring ROP (20).

There are variable trends in ROP outcomes in different geographic areas. Trends in increased neonatal mortality amongst Alaskan Natives, another darkly pigmented population, demonstrated increased susceptibility to ROP and more severe ROP amongst Native populations compared to non-natives (21–23). Townsel et al. performed a retrospective analysis of over 4,000 infants admitted to a NICU in Connecticut and found that Hispanic neonates experienced 70% more ROP and mixed-race neonates experienced 55% less ROP. Though Black and mixed neonates were more likely to have Medicaid in this study, the primary and secondary outcomes of the study remained unchanged after controlling for Medicaid status (24). Many studies have suggested that black infants have a lower risk for severe ROP (18, 20, 25), and are also more likely to be born premature (7–9). Future studies are needed to understand if this finding is due to increased black infant mortality prior to ROP screening, or if there are other variables affecting ROP severity in infants of color.

Socioeconomic status, geography, and race/ethnicity play crucial roles in health care access and utilization. Black and Hispanic families are known to be more affected by housing instability, longstanding residential segregation, systemic racism, and food insecurity which adversely affect child health (26, 27). Additionally, studies have demonstrated racial and ethnic differences in rates of necrotizing enterocolitis (NEC), bronchopulmonary dysplasia (BPD), intraventricular hemorrhage (IVH), and sepsis which are sequelae of prematurity (28–31). The transition from the inpatient setting to the outpatient clinic setting for ROP screening and follow up is a vulnerable period during which loss to follow up can lead to devastating visual outcomes. Identifying barriers to follow up in the outpatient setting is crucial as part of discharge and care planning, and requires the neonatology and ophthalmology teams to coordinate strategies to address these challenges prior to discharge.

In the sections below, we will discuss barriers to outpatient ROP follow up including access to care, high healthcare utilization, high readmission rates, patient health literacy, language barriers, social support and parental mental health.

### Access to Care

Insurance coverage has been shown to correlate with improved vision and eye health outcomes and lower rates of vision impairment (32). However, simply having health insurance does not equalize health care utilization or health outcomes across different racial/ethnic groups (33).

Wang et al. conducted a series of focus groups and interviews of parents and found that respondents' ability to manage their children's health care was limited by parental understanding of ROP, feeling overwhelmed by the infant's care, and unmet needs for resources to address social stressors. Other challenges include access to ophthalmologists with expertise in ROP care, coordinating and attending multiple outpatient appointments across different medical specialties, and lack of transportation (34). Even at sites with a tracking database and ROP coordinator, it was noted to be time and resource-intensive to ensure that exams and treatments were being performed according to protocol. Similar findings were noted in another study surveying 131 parents with preterm infants where it was found that the most common access barriers to attending ROP appointments reported by parents included few available ophthalmologists with expertise in ROP care (23%) and social situations (i.e., housing, transportation, or childcare) (22%) (35).

### High Healthcare Utilization

#### Multiple Clinic Visits

Healthcare utilization for families of preterm infants in the first 2 years of life is extremely high with increased clinic visits, hospitalizations, and medication usage (36–39). Most readmissions and use of healthcare services occur in the first weeks and months after initial NICU discharge (40–44). In addition to ocular sequelae, these infants are at risk for many other preterm comorbidities. These include BPD, NEC, IVH, patent ductus arteriosus, among others. These infants are also at risk for longer term sequelae of prematurity including neurologic abnormalities, developmental delays, and functional delays (45–47). Many of these diagnoses require multiple outpatient subspecialist follow up visits, which is a burden on the families who may also struggle with financial and employment insecurity due to missed work and loss of income (48).

The American Academy of Pediatrics recommends a minimum of seven well child care (WCC) visits in the first year of an infant's life (49). One study found that preterm infants average 20 or more visits to the doctor (WCC or specialty follow up), and preterm infants with multiple comorbidities averaged >30 visits per year (37). Twenty-five percent of these visits were for respiratory symptoms, growth, development, infections, or ROP follow-up.

In a study surveying parents of premature infants, 17% reported that having a large number of different appointments related to their infants' care was a barrier to ROP appointment follow up (35). The enormous amount of medical care for preterm infants after NICU discharge is an important barrier to follow up that should be addressed. Disparities in outpatient follow up rates have been noted, with lower attendance in post-discharge subspecialty care which occurs more often among Black families compared to White families (50, 51). Black infants were also more likely to miss their ROP follow up appointments than White infants (51).

#### High Readmission Rates

Preterm infants are a high risk population for readmission following discharge from the NICU (52–54). Morris et al. examined rehospitalizations of 1,591 extremely low birth weight infants from 14 centers of the National Institute of Child Health and Human Development Neonatal Research Network and found that 49% of these infants were readmitted before 18 months corrected age with respiratory, surgery, and infection listed as the top three causes (53). A larger study of 263,000 infants born between 1992 and 2000 in California found that readmission rates were much lower at 15%, but found similarly that the top causes of readmission were respiratory complications and infection (54).

Other studies have found racial, ethnic, and maternal age-related disparities in readmission rates (38, 42). In one study, compared to white infants, Hispanic and Black preterm infants were more likely to be readmitted in the first year of life (38). Additionally, infants born to mothers 19 years or younger had significantly increased odds of medical rehospitalizations and emergency department visits during the first 3 months after initial discharge compared with preterm infants born to young adult mothers (20–29 years of age) (42). This data suggests that identifying patients with these risk factors for high readmission rates should be considered by all members of the patients' care team and appropriate resources for support provided in order to ensure the best health outcomes for the child.

### Patient Health Literacy

Healthy People 2010 defines health literacy as “the degree to which individuals have the capacity to obtain, process, and understand basic health information and services needed to make appropriate health decisions” (55). Baker et al. found that even when socioeconomic status and baseline health status are accounted for, poor health literacy is associated with increased mortality (56). As described by the definition above there are many important components to patient health literacy such as provider communication, language barriers, and the patient and family's internalization of their disease process. Adverse outcomes associated with ineffective communication among any of these components can include misunderstandings of a patient's concerns, misdiagnosis, unnecessary testing, poor adherence, and inappropriate follow-up.

The American Medical Association and National Institutes of Health recommend that written resources be written at 3rd to 7^th^ grade level. John et al. analyzed articles for common pediatric ophthalmology conditions available online *via* search engine, including those available on professional society websites, and found that the majority of articles were written above recommended guidelines (57). In a prospective observational study of medication adherence in pediatric glaucoma, Freedman et al. utilized the Rapid Assessment of Adult Literacy in Medicine, a word recognition test that involves words commonly used in the health care setting, and found that decreased adherence was associated with lower health literacy (58).

It is also important to consider the role of recognizing parents' health literacy at the time of diagnosis, and to utilize health communication techniques when counseling parents. Eneriz-Wiemer et al. noted in a study of four California NICUs that only half of parents with preterm infants in the NICU reported receiving information about their infants' ROP status at discharge (35). This finding raises the question of whether providers failed to discuss the diagnosis or if the diagnosis was not understood by families, and emphasizes the importance of using effective health communication techniques.

### Language Barriers

Limited English proficiency (LEP) describes individuals who do not speak English as their primary language and who have a limited ability to read, speak, write, or understand English (59). In the United States, 30% of people are considered LEP (60), and patients with LEP have been shown to report poor communication with their provider, receive lower quality of care, have higher rates of misdiagnoses, and have significantly more emergency department visits and rehospitalizations (61–68). Parents are essential partners for ensuring timely outpatient follow up for ROP care. Studies have shown that LEP parents are more likely to report poor communication with health care providers compared to English proficient parents (65–67). Eneriz-Wiemer et al. found that among parents with premature infants, regardless of parent literacy level, parents with LEP had significantly lower odds of knowing that ROP is an eye disease of premature infants compared to literate, English-proficient parents (35).

Health care quality and outcomes improve for LEP patients and families when professional interpreters are used or language-concordant providers are available (69, 70). Unfortunately, it has been shown that LEP patients and families do not receive appropriate language services. A retrospective cohort analysis in an academic hospital showed that 66% of patients with LEP never had documentation of interpreter use during their hospital stay (71). Palau et al. conducted a structured interview of 132 English-speaking vs. Spanish-speaking parents in a Colorado NICU and found that NICU providers provided updates to Spanish-speaking parents in their native language only 39% of the time. In this same study, Spanish-speaking parents were four times more likely to incorrectly identify their child's diagnosis than English-speaking parents (72). These findings show that there are language-related health disparities that may be addressed to improve ROP care for patients with LEP.

### Social Support and Parental Mental Health

For parents with multiple children, childcare is essential to ensure that parents can attend outpatient ROP follow up, particularly in the COVID-19 era where multiple family members are often not allowed in the waiting rooms to reduce crowding. In a survey done among 71 parents who had an infant who required outpatient ROP care in California, 22% reported that childcare was amongst the barriers to attending ROP appointments (35). In another study by Miguel-Verges et al. interviewing 45 Latinx families, 28% reported that they had no primary support person in the US to help with childcare (73).

Parental mental health is an important component to ensuring appropriate ROP follow up. Parenting premature infants is a difficult and demanding task for parents (74–76). Parents report feelings of guilt and grief with the perceived loss of their “normal” child (77). This grief can lead to depression for one or both parents. Postpartum depression (PPD) has also been shown to occur more frequently in women of lower socioeconomic status (78). Yonkers et al. found that half of postpartum major depressive episodes begin prior to delivery (79) indicating that PPD may impact parental functioning prior to parents arriving to the NICU with their infant. Given that depressive symptoms include fatigue, loss of energy, loss of interest, hypersomnia, and feelings of guilt, PPD may be another barrier to health literacy and comprehension of physician counseling during outpatient follow up visits for ROP. In a focus group with 47 parents of very low birth weight infants, psychosocial stressors such as feeling overwhelmed by the infant's care, and unmet needs for resources to get to or pay for appointments were noted as factors in preventing these parents from taking their infants to outpatient ROP appointments (34).

### Patient-Oriented Solutions and Future Directions

Solutions to address the barriers to outpatient ROP follow up must consider the child in the setting of his or her environment and utilize a multidisciplinary approach that includes not only the ophthalmologist, but also ROP care coordinators, neonatologists, pediatricians, social workers, health communications workers and other healthcare providers. As outlined above, identifying risk factors such as access to care, health literacy, family and parental stressors, and health communication challenges, while recognizing the challenges specifically faced by minority populations in which disparities have been identified. Support for these interventions must be prioritized in policy setting at the local and national level. In the sections below, we proposed a series of patient-oriented solutions and future directions. Table 1 summarizes the potential barriers to outpatient ROP follow up. Table 2 highlights specific interventions that address the potential barriers of care described above.

**Table 1:** Potential barriers to outpatient ROP follow up.

**Potential barrier to follow up**	**Significant findings**	**References**
Access to care	1.Insurance 2.Lack of Transportation 3.Few ROP Specialists within distance	(34, 35)
High healthcare utilization *a. High number of follow up clinic visits b. High readmission rates*	1.Multiple outpatient subspecialist follow up visits 2.There are racial and ethnic disparities in clinic follow up rates for ROP 3.Readmission rates for premature infants are high and may affect ROP follow up rates 4.There are racial, ethnic, and age related disparities for outpatient clinic follow up rates	(35, 37, 38, 42, 51, 54)
Patient health literacy	1.Majority of online pediatric ophthalmology resources for the public are written above recommended reading level guidelines2. Decreased adherence for pediatric glaucoma patients was associated with lower parental health literacy 3.Only half of parents with preterm infants in the NICU reported receiving information about their infants' ROP status at discharge.	(35, 57, 58)
Language barriers	1.Regardless of parent literacy level, parents with limited English proficiency had significantly lower odds of knowing that ROP is an eye disease of premature infants 2.NICU providers provided updates to Spanish-speaking parents in their native language only 39% of the time 3.There are language related disparities that may be addressed to improve ROP care	(35, 71, 72)
Social support and parental mental health	1.Lack of childcare 2.Psychosocial stressors such as feeling overwhelmed by the infant's care, and unmet needs for resources to get to or pay for appointments 3.Postpartum depression (PPD) occurs frequently in women of lower socioeconomic status. PPD may be another barrier to health literacy and comprehension of physician counseling during outpatient follow up visits for ROP	(35, 73, 78, 79)

**Table 2:** Patient-oriented solutions to improve outpatient ROP follow up.

**Barrier to care**	**Patient-focused intervention**
High healthcare utilization (multiple follow-up visits, high readmission rates)	1. Utilizing ROP care coordinators to schedule follow-up visits on the same day as other visits 2. Creating tracking systems to monitor post-discharge care 3. Coordinating with inpatient providers to schedule ROP screening visits inpatient during readmission
Health literacy and limited English proficiency	1. Providing patients with ROP informational resources at recommended reading levels 2. Utilizing health communication techniques to confirm family's comprehension of physician counseling 3. Standardized counseling by ROP coordinators during outpatient clinic visits 4. Utilization of interpreter services at all visits in the inpatient and outpatient setting
Access to care	1. Utilization of social work to coordinate transportation and childcare to enable parents to attend follow up visits 2. Coordination with financial services to determine which providers are in-network and at minimal cost to patient
Social support and parental mental health	1.Engaging primary care physicians and mental health providers to proactively screen for postpartum depression and parental mental health

### Standardizing Personnel and Roles

#### ROP Coordinators

In high income countries, ROP coordinators are considered to be standard practice in the ROP care continuum to improve care coordination and follow-up. While the role of this coordinator is well-recognized, there are few standardized protocols, outcome and performance metrics, and training for this role to measure care outcomes and the effectiveness of the role. Wang et al. conducted an interview with 28 ROP providers (ophthalmologists, nurses, coordinators) who stressed the importance of the ROP coordinator role (34). The coordinator was able to build rapport with the families which was thought to encourage attendance at follow up ROP appointments, though the role of these subjective characteristics are more difficult to elucidate.

The Ophthalmic Mutual Insurance Company (OMIC) provides a toolkit for ROP care that outlines recommended protocols for screening, diagnosis, management, and care coordination (80). The OMIC Safety Net tool describes the role of both a hospital ROP coordinator (H-ROPC) and an outpatient ROP coordinator (O-ROPC). At the time of initial diagnosis, the role of the H-ROPC is to maintain the screening list, track infants, and work with the O-ROPC at discharge planning. The role of the O-ROPC includes scheduling the initial outpatient visit, coordinating transitions between outpatient ophthalmologists including the retina specialists and pediatric ophthalmologists, provide ongoing education, and manage a reminder system to ensure follow up.

#### Social Workers

Recognizing that families of patients with ROP are navigating multiple follow up appointments in addition to social and financial barriers, creating a team of healthcare providers to support the family may improve adherence. A social worker with expertise in pediatric care would be able to provide education and resources that take into consideration a family's individual social, behavioral, and cultural factors. Patients should be screened with a formal assessment for any barriers to care (cost, insurance, transportation, distance, housing insecurity, food insecurity, childcare) in the NICU before discharge and at their first ROP appointment so they can be provided with the appropriate resources. The success of social work intervention in pediatric screening programs has been demonstrated. In a study involving social workers in an inner-city vision outreach program, after the inclusion of a social worker, the follow-up rates for positive screenings increased from <5% to 59% (of 96 participants who required follow-up) (81). Silverstein et al. assessed the follow-up patterns of children referred for eye examination following a school based vision screening program, and offered social worker services and financial support to enable referred children to complete the eye examination (82).

Lack of transportation has been recognized as a barrier to ROP follow up as noted in multiple studies (26, 27). In a study cohort of very low birth weight infants, Catlett et al. were able to substantially increase developmental clinic attendance by providing transportation which was utilized in 31% of families (83). Providing transportation or reimbursement is a potential strategy to mitigate the barrier of transportation especially for families who travel significant distances. Although not specifically designed for children with ROP, addressing transportation challenges was identified as an area for further development in the Wills Eye Vision Screening Program for Children to improve follow up, Silverstein et al. described methods used to incentivize referral visits for eye exams, such as the provision of transportation tokens (82). This is one example of a specific area where social workers can provide ancillary support.

### Monitoring Post-discharge Care

Building on the previous discussion of the role of ROP coordinators, the second intervention is scheduling and monitoring post-discharge care, as well as ensuring that parents have the tools to communicate with the healthcare team. A key point is ensuring ROP follow up appointments are scheduled prior to NICU discharge and coordinating this with the ophthalmologists' staff and ROP coordinator. The effect of scheduling an appointment before discharge has been shown in many prior studies. In a randomized trial of pediatric asthma patients seen in a large urban emergency department, patients were significantly more likely to follow up with their primary care physician (64% vs. 46%) when an appointment was made for them at the time of the emergency department visit (84). A prospective study of 111 patients discharged from a pediatric intensive care unit found that compliance with subspecialty follow-up was significantly higher when follow-up was scheduled for a family vs. recommended (92% vs. 67%) (85). Attar et al. found that preterm infants at risk for ROP were much more likely to complete ROP follow up when it was scheduled for them prior to discharge (86).

### Health Communication Interventions

#### Use of Technology to Increase Follow Up

At the time of NICU discharge it is also important to assist families in enrolling in the electronic health record to utilize integrated communication platforms which enables parents to stay in contact with the healthcare team. Additionally, ensuring that telephone numbers as recorded in the patient chart are accurate so that telephone reminders can reach the appropriate family members. One study showed that 79% of parents surveyed reported telephone reminders were helpful at prompting attendance for their child's appointment (87). It is also important to consider the timing of telephone reminders when parents must juggle multiple commitments; while many reminders are provided 48 hrs in advance, phone calls placed more than 1 week in advance allowed families time to arrange for transportation, child care, and work coverage (88).

Due to the increase in smartphone use in our society, health systems are expanding their use of mobile Health (mHealth) to improve communication with patients, especially text messaging. The National Institutes of Health defines mHealth as “the use of mobile and wireless devices (cell phones, tablets, etc.) to improve health outcomes, health care services, and health research (89)”. There has been some variability in the evidence supporting the use of mHealth and text messaging to increase patient adherence to follow up. Tofighi et al. used text messaging to send appointment reminders to patients in an outpatient buprenorphine treatment program with 95% of patients reporting that text messaging was effective and favored over receiving telephone call reminders (90). Another study eliciting 50 parents opinions on text messaging showed that 100% of parents were willing to receive text messages for immunization reminders for their children (91). In a study done in Urology, a mHealth reminder, education program, and procedure preparedness assessment was created for patients to schedule a transrectal prostate biopsy. They found that in the post intervention cohort there were significantly fewer canceled or rescheduled appointments (33.8 vs. 21.2%), fewer same-day cancellations (3.8 vs. 0.5%), and increased patient satisfaction (4.5/5) (92). On the other hand, in a randomized controlled trial of 543 caregiver/child dyads using voicemail reminders vs. text messaging in a dental pediatric clinic, text messages were not as effective as voice reminders in increasing outpatient follow up (93). Future studies are needed to understand if using text messaging reminders or mHealth technology would be useful for parents of ROP infants to increase follow up adherence.

#### Improving Health Literacy and Language Barriers

Given the role of health literacy in improving communication between providers and families, the provision of health literature at an appropriate level is important for families to understand ROP and follow up screening and treatment. OMIC published a template that providers may use to standardize their approach in the hospital and in clinic on how to discuss ROP with families diagnosed in the NICU and a contract explaining the benefits and risks of treatment (80).

For families with LEP, it is necessary to increase professional medical interpreter use. Providers often believe that they spend more time with LEP patients than English-speaking patients even though it has been shown that there are no differences in appointment durations when professional on-site interpreters are used (94, 95). In a semi-structured interview conducted with 39 healthcare professionals from five specialties, providers reported that interpreters' ability to redirect and guide patients in the communicative process is valuable for time management (96). Telephone interpreter services (TIS) are useful and more accessible for healthcare providers especially now during the COVID-19 era. In a study interviewing 13 LEP patients, participants reported that TIS allowed them access to physicians with whom they felt the most comfortable, regardless of language ability and provided patients reassurance of accurate communication about their medical care (97). More research is needed to assess the use of interpreters and the reasons why ophthalmologists may not use interpreters in ROP clinics to better understand what solutions are needed.

## Conclusions

This review article highlights the complexity of ROP care, and the need for a team-based approach to identify and address barriers to care including access to care, health literacy, language barriers, high healthcare utilization, and frequent need for follow-up. Parents of infants with ROP often face a multitude of social, emotional, and financial factors that can affect adherence to care, and it is important for not only ophthalmologists, but the entire care team, to recognize and align resources to provide the best care possible to prevent vision loss.

## Author Contributions

TN, EC, AS, and RC: conception and design. TN, EC, and MChe: data collection and literature review. TN, EC, and RC: analysis, interpretation, and drafting of manuscript. TN, EC, AS, MChi, JC, and RC: critical revision of manuscript and overall responsibility. RC: obtained funding. All authors contributed to the article and approved the submitted version.

## Funding

This project was supported by R01EY029673, R01EY19474, K12 EY021475 from the National Eye Institute, National Institutes of Health (Bethesda, MD; Grant No. P30 EY10572) and by unrestricted departmental funding from Research to Prevent Blindness (New York, NY). The sponsor or funding organizations had no role in the design or conduct of this research.

## Conflict of Interest

The authors declare that the research was conducted in the absence of any commercial or financial relationships that could be construed as a potential conflict of interest.

## Publisher's Note

All claims expressed in this article are solely those of the authors and do not necessarily represent those of their affiliated organizations, or those of the publisher, the editors and the reviewers. Any product that may be evaluated in this article, or claim that may be made by its manufacturer, is not guaranteed or endorsed by the publisher.
